# Supplementary material for: A Humanized Yeast Phenomic Model of Deoxycytidine Kinase to Predict Genetic Buffering of Nucleoside Analog Cytotoxicity
Source: Genes (Basel). 2019 Sep 30;10(10):770. doi: 10.3390/genes10100770 (PMC6826991; doi:10.3390/genes10100770)
Supplement: Supplementary file 1 [file genes-10-00770-s001.zip › Additional_Files/Additional_File7_GOTermHeatmaps.zip!/Additional_File7_GOTermHeatmaps/heteroduplex_formation.pdf]

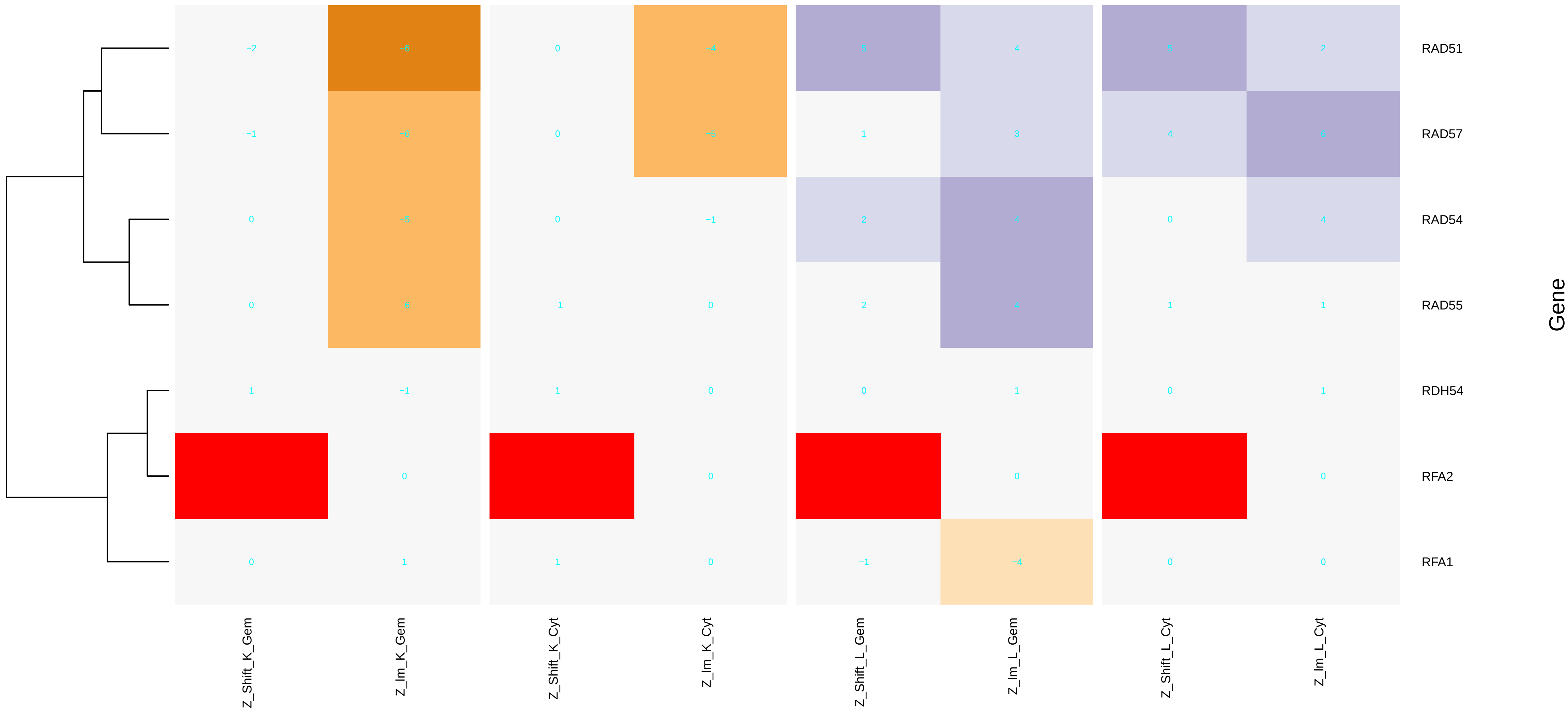
Gene
RAD51
RAD57
RAD54
RAD55
RDH54
RFA2
RFA1
Z_Shift_K_Gem
Z_Im_K_Gem
Z_Shift_K_Cyt
Z_Im_K_Cyt
Z_Shift_L_Gem
Z_Im_L_Gem
Z_Shift_L_Cyt
Z_Im_L_Cyt
−2 −6 0 −4 5 4 5 2
−1 −6 0 −5 1 3 4 6
0 −5 0 −1 2 4 0 4
0 −6 −1 0 2 4 1 1
1 −1 1 0 0 1 0 1
0 0 0 0
0 1 1 0 −1 −4 0 0
